# Effectiveness of a Locally Produced, Fish-Based Food Product on Weight Gain among Cambodian Children in the Treatment of Acute Malnutrition: A Randomized Controlled Trial

**DOI:** 10.3390/nu10070909

**Published:** 2018-07-16

**Authors:** Sanne Sigh, Nanna Roos, Chhoun Chamnan, Arnaud Laillou, Sophanneary Prak, Frank T. Wieringa

**Affiliations:** 1 Department of Nutrition, Exercise, and Sports, Faculty of Science, University of Copenhagen, Rolighedsvej 26, 1958 Frederiksberg C, Denmark; nro@nexs.ku.dk; 2 Department of Fisheries Post-Harvest Technologies and Quality Control, Fisheries Administration, 186 Preah Norodom Boulevard, Phnom Penh 12101, Cambodia; chhounchamnan@gmail.com; 3 United Nations Children’s Fund Cambodia, Department of Child Survival and Development, 19 & 20, Street 106, Exchange Square Building, Phnom Penh 12101, Cambodia; alaillou@unicef.org; 4 National Nutrition Program, Ministry of Health, 31A Rue de France (St. 47), Phnom Penh 12202, Cambodia; sophonprak@gmail.com; 5 UMR-204, Institut de Recherche pour le Développement, IRD/Université de Montpellier/SupAgro, 911, avenue d’Agropolis, 34394 CEDEX 5 Montpellier, France; franck.wieringa@ird.fr

**Keywords:** severe acute malnutrition, fish, ready-to-use therapeutic foods, effectiveness, weight gain, Cambodia

## Abstract

Cambodia continues to have a high prevalence of acute malnutrition. Low acceptability has been found for standard ready-to-use-therapeutic-food (RUTF) products. Therefore, NumTrey, a locally-produced fish-based RUTF, was developed. The objective was to evaluate the effectiveness of NumTrey compared to an imported milk-based RUTF for weight gain among children aged 6–59 months in the home-treatment for acute malnutrition. Effectiveness was tested in a single-blinded randomized controlled trial with weight gain as the primary outcome. Anthropometry was assessed at baseline and bi-weekly follow-ups until endline at Week 8. In total, 121 patients were randomized into BP-100^TM^ (*n* = 61) or NumTrey (*n* = 60). There was no statistical difference in mean weight gain between the groups (1.06 g/kg/day; 95% CI (0.72, 1.41) and 1.08 g/kg/day; 95% CI (0.75, 1.41) for BP-100™ and NumTrey, respectively). In addition, no statistically significant differences in secondary outcomes were found. Although the ability to draw conclusions was limited by lower weight gain than the desired 4 g/kg/day in both groups, no superiority was found for eitherRUTF. A locally produced RUTF is highly relevant to improve nutrition interventions in Cambodia. A locally produced fish-based RUTF is a relevant alternative to imported milk-based RUTF for the treatment of SAM in Cambodia.

## 1. Introduction

Globally, it is estimated that 51.5 million children below 5 years of age are suffering from moderate acute malnutrition (MAM) and additionally 18.7 million children from severe acute malnutrition (SAM) annually [1]. MAM is defined as a weight-for-height z-score (WHZ) between −2 and −3, and/or a mid-upper-arm circumference (MUAC) between 115 and 125 mm. SAM is defined as WHZ <−3, and/or a MUAC <115 mm, and/or the presence of nutritional edema regardless of anthropometric indices [2].

Despite improvements in food security and reduction in poverty rates in Cambodia [3], the latest National Demographic Health Survey in 2014 showed that, among preschool children, 12% were diagnosed with acute malnutrition (SAM and MAM combined). The prevalence of acute malnutrition has stagnated since 2005 [4]. Besides a retrospective review of clinical records from 1999 to 2006 [5], no studies have been conducted on outpatient treatment of SAM in Cambodia. In the Cambodian health system and in the National Demographic Health Surveys, children are recorded by the classification WHZ <−3, therefore the exact degree of SAM is unknown.

Children with SAM require immediate treatment with specialized therapeutic diets and hospitalization if complications are present [5,6,7]. Hospital-based treatment is at the expense of work and family, and it also predisposes the children to hospital-acquired infections [7]. Children with SAM without complications can be treated as outpatient with home-based treatment.

The introduction of the community-based management of acute malnutrition (CMAM) using ready-to-use-foods (RUTFs) made a major improvement in the survival of children suffering from non-complicated SAM [8,9,10] and contributed to overcoming the problems that arise from hospitalization [11]. A RUTF is a nutrient- and energy-dense product, which according to the current international specifications, must contain milk products supplying at least 50% of overall protein, along with added vitamins and minerals [10].

Commonly used RUTF products in SAM treatment are Plumpy’Nut^®^ and BP-100™, produced by companies in Europe. In Cambodia, Plumpy’Nut^®^ was found to have low acceptability [12] and therefore the BP-100™ biscuit is currently used by UNICEF (Phnom Penh, Cambodia) and the Cambodian government [13]. However, an unpublished survey indicated that BP-100™ may also have modest acceptability [14], and SAM treatment in Cambodia could possibly be improved if products adapted to local preferences were available. Therefore, in 2013, we initiated the development of a locally produced RUTF based on locally available ingredients: rice, soybean, mung bean, canola oil and small indigenous fish. Fish is seasonally highly available and is a sustainable source of protein in Cambodia. Small-sized fish are particularly nutritious [15], and could be a cheaper alternative to milk powder in RUTF products. Small dried, powdered fish has been shown to be a possible alternative to milk in fortified blended food products [16].

The overall aim of the project was to develop a RUTF accepted by the target group which could support successful treatment of SAM in Cambodia. Our initial acceptability taste trial showed that a pure fish-based paste product was not well accepted, but when filled into a crispy wafer, the fish-based paste became more acceptable among children and their caregivers [17]. Acceptability, compliance, and sharing from the effectiveness trial reported here were also described [17]. 

Assessment of efficacy and effectiveness of locally made RUTFs, using commercially produced RUTFs as the comparison is recommended by the World Health Organization (WHO) [18]. Therefore, the effectiveness of the local fish-based wafer product in the treatment of SAM needed to be confirmed to support the decision of replacing the imported milk-based RUTF in Cambodia.

The present trial was conducted to evaluate the effectiveness of the locally produced fish-based wafer RUTF (named “NumTrey”) compared to the imported milk-based RUTF (named “BP-100™”) for weight gain during outpatient treatment of acute malnutrition among Cambodian children age 6–59 months.

## 2. Materials and Methods

### 2.1. Trial Setting

The trial was conducted at the National Pediatric Hospital in Phnom Penh, Cambodia, between 7 September 2015 and 7 January 2017, among patients admitted to the outpatient department for treatment of SAM. On 7 January 2017, the last patient finished his/her eight-week treatment. The hospital has a standard protocol for the treatment of SAM patients with the presence of complications in place.

### 2.2. Trial Population

All patients diagnosed with SAM without complications aged 6–59 months were eligible for the trial. The SAM patients had either started their SAM treatment as an inpatient where complications were treated before discharge to continue outpatient treatment of SAM or were directly referred to the outpatient treatment of SAM at the outpatient department. If the patient had received inpatient treatment to treat complications and SAM, the assessment of diagnostic criteria was conducted immediately after discharged from inpatient treatment.

In general, CMAM trials could either receive patients treated as inpatient prior to inclusion or directly referred to CMAM, resulting in some patients being close to the SAM/MAM border at baseline. To ensure these patients were eligible, the cutoff used as inclusion criteria were adjusted. Hence, patients who have been treated as inpatient prior to enrolment of the trial were eligible; the diagnostic criteria for SAM to be enrolled in the present trial was set at WHZ ≤ −2.8 and/or MUAC ≤ 115 mm, and/or presence of nutritional edema. Consequently, recovery rate as the main outcome could not be used. To prevent confusion on whether a child was diagnosed with SAM or MAM, we used weight gain as the primary outcome and as continues variable (increase in weight gain (g)), and changes in WHZ and MUAC as secondary outcomes.

Before inclusion of eligible patients in the trial, the patients had to pass an appetite test and the caregivers have to sign an informed consent. Exclusion criteria were uncontrolled or untreatable systemic opportunistic infection, severe cerebral palsy, obvious dysmorphic features, general mental health problems, or participation in other clinical trials. Human immunodeficiency virus (HIV) and tuberculosis infections were not considered exclusion criteria. Patients who developed complications such as fever and diarrhea during the intervention period remained in the trial.

### 2.3. Ethical Considerations

The trial was approved by the National Ethical Committee for Health Research of the Ministry of Health, Kingdom of Cambodia (April 2015 Version No. 2) and was conducted in accordance with the ethical standards in the Declaration of Helsinki.

At the time of recruitment, caregivers were informed about the purpose of the trial, the allocated RUTFs, and each step of the data collection. Written and oral informed consent was obtained from the patient’s parent or legal guardian before the patient was enrolled in the trial. Patients who refused to eat the RUTF they were allocated for more than four days/week over two consecutive weeks or patients who lost weight during two consecutive visits were either offered the other RUTF (if they passed the appetite test) or another appropriate action was taken (depending on the patient and feasibility). Their intakes were still included in the final analysis of the trial.

All participants were compensated for their time with a small gift after finishing the trial. The trial is registered at ClinicalTrials.gov (Trial name: “Comparison of a Locally Produced RUTF with a Commercial RUTF in the Treatment of SAM (FLNS_SAM)”, trial registration; NCT02907424).

### 2.4. Trial Design

This trial was a prospective, randomized, single-blinded, home-based trial with a 1:1 allocation ratio (ratio of intended numbers of participants in each group) designed to assess weight gain as the primary outcome. After recruitment, the patients were randomly allocated to one of the two interventions: (1) standard treatment with BP-100™ (active control); or (2) treatment with the locally produced fish-based RUTF NumTrey (intervention). The duration of the intervention was eight continuous weeks, with follow-up visits every two weeks.

The RUTFs and the packaging were visibly different from each other, therefore, the blinding of hospital staff, participants, and the project staff responsible for outcome measures was logistically not possible. Product codes (X or Z) replaced the names of the two RUTFs in all data registrations. The codes were prepared to blind the researcher (S.S) who was responsible for the trial. The researcher (S.S) supervised the project staff during the trial, cleaned the data and conducted the data analysis after the trial. The primary analysis of data had been completed before the code was provided to her.

A computer-generated randomization list in blocks of four patients based on the product codes and patient ID number was made prior to the start of the trial using http://www.randomization.com/ by one of the principal investigators. The list was provided in a closed envelope to the project manager, who enrolled participants and assigned the intervention to the participants based on the list.

An appetite test is recommended by WHO to ensure that the patient had the appetite to eat the RUTF to which the patient was allocated [18,19]. The test shows whether the child has sufficient appetite, accepts the RUTFs taste and consistency and can swallow the RUTF. Appetite was assessed by asking the caregiver to sit quietly for as long as it takes (usually ~15 min) with the patient while actively offering the RUTF. The patients were offered water during the appetite test. Generally, the patient is offered 1 sachets of RUTF (92 g) and the patients have to eat at least 1/3 [19] independent of age 6–59 months. However, a 6-month-old patient and a 58-month-old patient would be expected to eat different amounts of RUTF, and therefore we developed age-dependent amounts to be eaten to pass the appetite test.

For patients 6–11 months, the minimum consumption was either 1 wafer (NumTrey) or 1/3 bar (BP-100™). For 12–23-, 24–35-, and 36–59-month-old children, the minimum consumption to pass the appetite test was 1.5 wafers or 1/3 bar, 2 wafers or 2/3 bar, and 3 wafers or 2/3 bar, respectively. The appetite test was evaluated based on the above criteria for minimum consumption by the interviewer.

**Figure 1:**
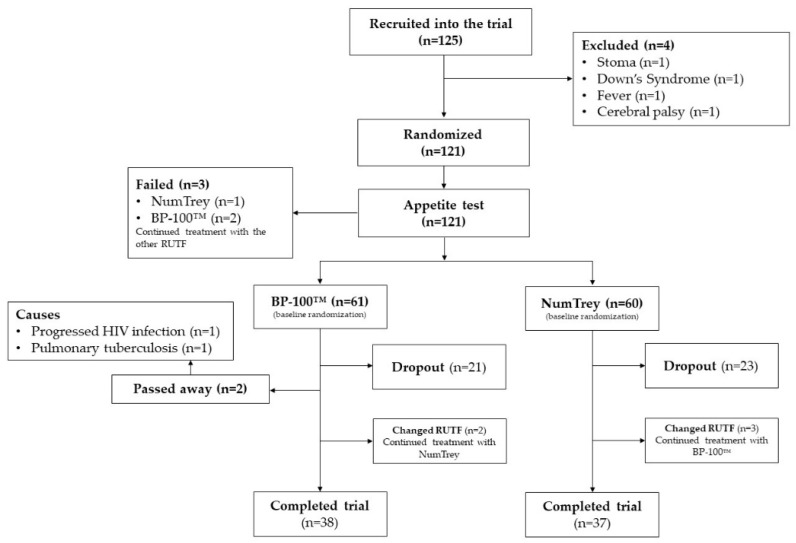
Flowchart of patient’s enrolment into the trial.

If the appetite test for the RUTF to which the child was randomly assigned failed, the child was offered the other RUTF to ensure the treatment of SAM was continued (see Figure 1). In cases where the patient failed the appetite test for both RUTFs, the patient was referred to inpatient treatment of SAM at the National Pediatric hospital. Patients who changed from one RUTF to the other RUTF during the trial remained in the trial since the trial primarily tested the effectiveness and not efficacy it was considered as unbiased for the trial.

### 2.5. Data Collection and Follow-Up

The outcome measures were collected by doctors and nurses employed by the National Pediatric hospital, as well as by nurses, midwives and master students in nutrition hired as project staff. All data were collected at the National Pediatric hospital. All data collected during the trial were entered directly into tablets using the application KoboCollect from where the forms were directly uploaded into an online digital survey tool (KoBoToolbox (Harvard University, Cambridge, MA, USA)). Using a digital data collection method has been found to reduce the cost, time, and errors compared to traditional collection methods [20,21].

All patients diagnosed with SAM at health centers, the hospital or at mass screenings were examined by one of the project staff and if any doubt confirmed by the responsible researcher (S.S).

The anthropometric measures obtained were: weight, length/height, and MUAC. All anthropometric measures were measured in triplicate by trained staff. The mean of the three measures was used for the statistical analysis. Weight was measured in light clothes (no diapers) to the nearest 100 g using a digital secamedical scale (model 872) (Hamburg, Germany). The length was measured for patients <24 months and height for patients ≥24 months using a standard wooden length board provided by UNICEF (Phnom Penh, Cambodia). Length/height was recorded to the nearest 1 mm. MUAC was measured using a standard MUAC tape (from UNICEF, Phnom Penh, Cambodia) to the nearest 1 mm on the patient’s left arm. Bilateral pitting edema was assessed by pressing a finger for 8–10 s on the foot, hand, and forearm. Facial and abdominal nutritional edema was diagnosed by a doctor at the National Pediatric hospital. The age of the patient was obtained from the growth and immunization cards provided at birth.

All patients participating in the trial were asked to return to the National Pediatric hospital for a follow-up visit every two weeks until they completed eight consecutive weeks. All anthropometric measures and edema, as well as medical complications, were assessed at each visit to the National Pediatric hospital.

### 2.6. Treatment Protocol

At the time of this trial, the discharge criteria for SAM children hospitalized as an inpatient was a weight gain of 15% since admission, while discharge criteria for home-based outpatient treatment was a MUAC > 115 mm [22]. The Cambodian SAM treatment protocol has since been updated [23,24,25]. Deworming treatment of SAM patients without complications is part of the standard treatment [22]. Therefore, all patients (in both RUTF groups) in the age groups 6–12 months, 12–23 months, and 24–59 months were provided 100 mg (single dose), 200 mg (single dose), or 400 mg × 3 days of albendazole (ZENTTEL^™^) (GlaxoSmithKline (gsk), Phnom Penh, Cambodia), respectively, at baseline. The total duration of the trial was eight weeks, which is the international standard duration of SAM treatment [6] and also the standard duration in Cambodia for outpatient treatment of SAM without complications [22]. Patients were provided with a two-week ration of RUTF based on weight at each follow-up visit. The patients were scheduled to come for follow-up every two weeks. In cases where the caregiver and patient did not attend the scheduled visit, project staff called and rescheduled a visit (if possible). The importance of follow-up visit was emphasized and encouraged and the caregiver was told that the RUTF was a “medicine” only for the patients and should not be shared. Nevertheless, due to the trial design being an effectiveness trial, it was accepted if the caregiver skipped one or more visits. Therefore, theoretically, a patient could attend the baseline visit and first return again for the last visit at endline.

The caregiver received all the necessary instructions regarding the products, nutritional education and was provided with contact information if they had any questions.

### 2.7. RUTFs Used in the Trial

The nutritional composition of the two RUTFs and comparison with the guidelines for the nutritional composition of RUTFs are presented in Table 1. The development and description of NumTrey have been reported elsewhere [17,26]. NumTrey has been tested for acceptability in a taste trial using a crossover design [17]. As shown in Table 1, the NumTrey paste fulfills the requirements for most macro- and micronutrients. When the paste was filled into the crispy wafer and the wafer was included, the nutrition composition, i.e., the ratio of macro- and micronutrients, decreased, thus not fulfilling all requirements for RUTFs. Therefore, during this trial, the patients were given a RUTF ration (depending on the patient’s weight) which was calculated based on the nutrition composition of the NumTrey paste alone (without the wafer). This resulted in distribution of a slightly higher food ration to patients receiving NumTrey. The food ration was calculated to between 160 and 180 kcal/kg for both RUTFs (Table 2).

The standard product BP-100™ is produced by the company Compact in Norway (Søfteland, Norway). Information on BP-100™’s nutritional composition was obtained from the label on the packaging.

### 2.8. Sample Size and Outcome of Interest

The main outcome of the trial was to compare the superiority of any of the two RUTFs regarding weight gain.

An average increase in body weight of 4 g/kg/day was the minimum desired gain based on WHO recommendations and on a home-based treatment study from Cambodia (2011) [5,27,28]. To detect a 10% difference, which was regarded as biologically relevant (that is, a difference in weight gain of >0.4 g/kg/day) and with a standard deviation (SD) of 0.7 g/kg/day, a sample size of 49 patients per intervention group was required (80% power; α = 0.05; two-sided). The 10% difference was based on a study conducted in Vietnam, comparing a locally produced RUTF with Plumpy’Nut^®^. In the study in Vietnam, a total weight gain of 1.2 kg (both groups) was reported with an SD of 0.6 and 0.5 in the two groups, respectively. However, the weight gain expressed as g/kg/day had a higher SD (1.3). The 10% difference as biologically relevant was chosen arbitrarily [29].

To allow for drop-out, it was planned to recruit 60 patients in each intervention group, thus a total of 120 patients. The desired weight gain ± SD was at least 4 ± 0.7 g/kg/day for both RUTFs [27,28].

As described above, the secondary outcomes of interest were: changes in total weight (g), height (cm), MUAC (mm), WHZ, weight-for-age z-score (WAZ), and height-for-age z-score (HAZ). All anthropometric measures were assessed every two weeks at follow-up visits, and z-scores were calculated.

### 2.9. Statistical Analysis

Data were entered using tablets and were uploaded to an online software system where the data collection forms were created. Data cleaning was done by reviewing all data variable to detect and correct (or remove) corrupt or inaccurate records, e.g., data typing errors of anthropometric measurements, date of birth, gender, product code, and age. Consistency between all data collection forms was checked. All basic information on the patient—date of birth, gender, anthropometric measures and product code—were noted in the field book for each follow-up visits. In cases where the data were inconsistent, the field book was checked for consistency between the field book and the data entry forms. If they were inconsistent, it was corrected according to the field book or for product codes the randomization list (a project staff did this to keep the blinding of S.S). No data were completely removed during the data cleaning.

Statistical analyses were carried out using the statistical software program R (version 3.4.0, Windows) [30]. In particular, the extension package lme4, multcomp, and lsmeans were used for the mixed-model analysis and the car package to calculate estimates of the main outcome. Anthropometric indices were calculated using WHO 2006 standards (ANTHRO version 3.2.2, January 2011, macro for R) expressed as z-scores for WHZ, WAZ, and HAZ.

Descriptive statistics are presented as percentages (*n*) or means (SD) for continuous variables. An analysis of the effectiveness of the intervention was based on the randomization of the product the patients were originally assigned using all available case data including patients missing follow-ups and dropouts [31]. This analysis method handles missing data by fitting a statistical model over all available case data without introducing bias.

Outcomes measured repeatedly at each visit (weight, height, MUAC, WHZ, WAZ, and HAZ) were analyzed using repeated measurements statistics to assess differences in growth patterns between the two RUTFs during the eight-week treatment using a linear mixed model. All dependent variables were analyzed by means of a linear mixed model. The models were analyzed with a time × treatment interaction; baseline measure of the variable was included in the model [32] as well as age, gender, and days (deprivations in days from the protocol with follow-up visits every two weeks) as fixed effects and subject-specific random effects. The results are presented as estimated means and SE, and a *p*-value for the difference between the two RUTFs.

The primary outcome weight gain as g/kg/day was estimated from the linear mixed model for weight estimates, including adjustment over the duration of the trial (56 days) and the difference between the two RUTFs. These results are presented as estimated means (SE), estimated differences in change from baseline between treatments (SE) and a *p*-value for the test of no difference or 95% CI.

Aside from the effectiveness analysis, a secondary analysis of efficacy was conducted by analyzing the patient’s actual treatment as consequence of treatment switch during the trial. This analysis was also based on available cases including missing follow-ups and dropouts. Model checking was based on visual inspection of residuals and normal probability plots. The sample size was too small for any meaningful interim analysis. A significance level of 0.05 was applied.

## 3. Results

### 3.1. Participants

The enrolment schedule of patients based on the randomized product is shown in Figure 1. Screening of patients was carried out between 7 September 2015 and 7 November 2016, and a total of 125 eligible patients were included. Four patients were excluded prior to randomization because of a stoma, Down’s syndrome, fever, or cerebral palsy. In total, 121 were randomized to either BP-100™ (*n* = 61) or NumTrey (*n* = 60). Out of 121 patients. Eight patients (four in each group) changed RUTF after randomization and continued treatment. Unfortunately, two patients receiving BP-100™ died during the trial. One patient died due to a progressed HIV infection and the other patient due to pulmonary tuberculosis.

The characteristics of the patients based on randomization at baseline are shown in Table 3. The analysis includes all patients enrolled in the trial. Overall, the patients in the NumTrey group were slightly older than the patients receiving BP-100™, with a mean age of 22.7 and 19.7 months, respectively. This is reflected in a higher prevalence (49.2%) of patients still being breastfed among patients receiving BP-100™ compared to NumTrey (45.0%). In addition, the mean weight and height of the patients in the BP-100™ are slightly lower compared to NumTrey. For the whole trial, the majority of the patients were male (58.6%).

### 3.2. The Effectiveness of the Intervention on Anthropometric Changes

#### 3.2.1. Primary Outcome Weight Gain g/kg/day

The estimated weight gain, as the primary outcome in the effectiveness analysis, based on available cases for BP-100™ during 56 days of treatment, was 1.06 g/kg/day; 95% CI (0.72, 1.41). For NumTrey, the weight gain was 1.08 g/kg/day; 95% CI (0.75, 1.41). There was no statistically significant difference between the two RUTFs, which was −0.02 g/kg/day; 95% CI (−0.49, 0.46).

The efficacy analysis, taking actual treatment into account, showed similar estimated weight gains. For BP-100™, the estimated weight gain in the efficacy analysis was 1.10 g/kg/day; 95% CI (0.76, 1.43), compared to an estimated weight gain for NumTrey of 1.03 g/kg/day; 95% CI (0.69, 1.37). The difference in change between the two RUTFs was not statistically significant (*p* > 0.05, difference 0.06 g/kg/day; 95% CI (−0.41, 0.54)).

#### 3.2.2. Analysis of Secondary Outcomes

In the effectiveness analysis of the secondary outcomes, there were also no statistically significant differences between the two RUTFs from baseline to any follow-up visit for weight-related anthropometric measures, i.e., total weight, WHZ and MUAC (Table 4).

In the efficacy analysis of the secondary outcomes, we found no statistically significant difference for total weight and MUAC, while a statistically significant difference for WHZ in favor of NumTrey was found only at the second follow-up visit (Table 4).

Height-related secondary anthropometric measures (total height and HAZ) are shown in File S1 (Table S1). A statistically significant difference was found for height gain in favor of BP-100^TM^ from baseline to endline in both the effectiveness analysis and the efficacy analysis. However, no statistically significant difference was found between the products for HAZ in both analyses.

## 4. Discussion

This trial did not find superiority in any of the two products in weight gain (g/kg/day). The trial demonstrated that a locally produced RUTF for the treatment of SAM, produced with small indigenous fish replacing milk as a protein source, might be as effective in terms of weight gain as an imported milked-based RUTF. Previous studies have used different indicators to assess the effectiveness and efficacy of RUTF products. Some studies used weight gain either as g/kg/day [7] or weight gain percentage [33]. Others used recovery [34,35,36,37,38,39,40], 100% of mean WHZ of the population [41], or combinations of two indicators, for example weight gain (g/kg/day) and recovery [42,43,44], weight gain and mortality [45], MUAC and weight gain [46] or more than two indicators [47,48,49]. Nutritional programs using MUAC recommended a weight gain of 15–20% [2], to avoid non-applicability of WHZ for children admitted based on MUAC who already fulfilled the WHZ discharge criteria at admission and to avoid using height measurements [2,27,50,51,52]. This leads to a smaller weight gain of the most malnourished children to meet the discharge criteria despite potential insufficient recovery from SAM [51,53]. Currently, the indicator used (WHZ or MUAC) at admission and discharge is recommended to be the same [18]. However, several studies have shown that MUAC and WHZ identify different sets of children having SAM [54,55,56]. Despite this awareness, no recommendations are currently made on the admission or discharge of children diagnosed with both indicators (MUAC <115 mm and WHZ <−3), which complicates outcomes measures in studies using both indicators, including the present trial. Therefore, we used weight gain as g/kg/day to avoid the potential risk of insufficient recovery from SAM by using weight gain percentages.

A weight gain of >4 g/kg/day for both RUTFs was the minimum desired in this trial [27,28]. Some studies have found weight gains ~4–5 g/kg/day [5,39,43,57] and up to ~9.5 g/kg/day for RUTFs in home-based treatment [43,58,59]. We found low weight gains just above 1 g/kg/day for both RUTFs, which also have been reported in other trials. One trial found rates of weight gain of 3.1 g/kg/day and 2.9 g/kg/day for whey-protein based and peanut-based RUTFs, respectively [42]. Another trial found weight gains of 2.8 ± 3.2 g/kg/day for the first four weeks of treatment, which is where the highest rates of weight gain are typically observed [60]. The WHO recommended a weight gain of minimum 4 g/kg/day [27,28] in the treatment of SAM was not reached in any of the above-mentioned studies, which mainly included patients directly from the community or outpatient departments. Thus, the patients had not received inpatient treatment of SAM prior to enrolment into the trials. Perhaps the WHO recommendations are too ambitious for these types of treatment programs. In the current trial, most patients were bordering the cutoff between SAM and MAM, meaning less weight gain per kg can be expected. It could be assumed that if only including patients with WHZ <−3, higher weight gains might have been seen. However, this was not confirmed by a post hoc analysis of patients with WHZ <−3 showing weight gains of 1.10 ± 0.25 g/kg/day (*n* = 24) for BP-100™ and 1.21 ± 0.27 g/kg/day (*n* = 25) for NumTrey with no statistically significant difference between the RUTFs (0.11 ± 0.37 g/kg/day 95% CI (−0.82, 0.60)). Although higher than in the overall group, this weight gain still falls short of the 4 g/kg/day. The modest weight gain indicates that outpatient SAM treatment could be improved in Cambodia. However, for outpatient treatment programs, the challenges of not being able to control all factors, e.g., compliance, quality of the meal given to the patient, feeding frequency, hygiene and sanitation, compared to in inpatient treatment might limit the impact of treatment.

This trial found no statistically significant difference in MUAC between the two RUTFs. The estimated MUAC gain was 0.09 mm/day (95% CI; 0.06, 0.12) and 0.05 mm/day (95% CI; 0.02, 0.08) for BP-100^TM^ and NumTrey, respectively, which were not statistically significantly different (0.04 ± 0.02 mm/day; 95% CI (−0.01, 0.08)) (post hoc analysis, data not shown). These MUAC gains are considerably lower than those reported in other trials, where MUAC gains of 0.3–0.37 mm/day was found [35,43,60]. This confirms that the Cambodian program for outpatient treatment is challenging and could be improved.

We found a small difference in WHZ change at a single follow-up visit in the efficacy analysis taking changes in treatment into account, in favor of NumTrey. The difference is inconclusive since the difference between the groups was not consistent across the whole treatment period.

Overall, this trial showed low rehabilitation from acute malnutrition in the outpatient treatment, in the standard as well as the experimental group. The weight gain in this trial was unfavorable to those reported by other CMAM programs where the average weight gains for children managed for acute malnutrition varied from 1.8 to 6.8 g/kg/day [42,46,60,61,62], and other studies in hospitalized children for treatment of SAM also found considerable higher rates of weight gain [7,9]. The lower weight gain in this trial could be caused by multiple factors, including the difference in adherence to an ideal management of patients with SAM, low daily feeding frequency, sharing, low acceptability and therefore a low overall consumption of RUTF. Although consumption was not directly assessed, acceptability, compliance, and sharing during the trial was assessed, which was reported in detail by Sigh et al. (2018) [17]. The proportion of patients expressing that they liked the product increased after the eight-week intervention for NumTrey and remained ~90% for BP-100^TM^ during the trial. The steady increase in acceptability of NumTrey can be seen as an adaption to an unfamiliar product, not uncommonly seen in acceptability testing of novel products [63].

Compliance was 48.1% and 51.7% for NumTrey and BP-100^TM^, respectively [17]. The compliance measure did not take into consideration whether the RUTF had been consumed by the patient or for example shared, sold or lost. When asking caregivers regarding sharing of the products, 46.7% (BP-100^TM^) and 64.6% (NumTrey) of all participants reported some sharing. The most reported shared, lost, wasted or sold was as a “small bites” among the categories; a small bite, half a bar/wafer, and 1 bar/wafer, however, do not have objective data on the amount of RUTF that was shared.

The reasons for more reported sharing of NumTrey could be that it is a new product, and it bears a resemblance to a local Cambodian snack sold in the market. Low compliance and sharing of RUTFs is a potential factor influencing the low weight gain in this trial.

Another important factor for weight gain is food consumption and calorie intake additional to RUTF. A recent study found that >70% of the children were not meeting the minimum acceptable diet in Cambodia [64]. Although no data were collected on food diversity or food quantity, this could influence the primary outcome of this trial.

Caregivers were strongly encouraged to come for follow-up visits. However, the caregivers were not always able to get permission to, e.g., leave work. Testing the effectiveness in a “real-life” situations needs to takes these into account. This resulted in some patients missing provision of RUTF during the intervention period, which likely influence the overall performance of the program.

The focus group discussions conducted with groups of caregivers indicated that caregiver prior to treatment lacked understand of that their child suffered from acute malnutrition.

It could be speculated the caregivers who do not fully understand the meaning and consequences of acute malnutrition would not necessarily feed the child with sufficient amounts of RUTF and as frequently as required for the patient to gain weight resulting in low weight gains. Contrarily, information, knowledge, and understanding do not necessary lead to behavior change or actions being taken to improve health situations [65,66].

### Strengths and Limitations

The trial experienced low attendance rate at follow-up visits and at endline, though similar in both groups at endline. In addition, our power calculation overestimated the effect of the trial resulting in a relatively low sample size combined with the high drop-out rate (38%). In most trials, a ~20% drop-out rate is anticipated, which was also done in this trial.

A statistical model using the available cases was selected to deal with the challenges of missing data during the analysis. The low attendance is probably caused by the trial design testing the effectiveness of the RUTFs, which aims to test the intervention as close to “real-world” circumstances as possible. An efficacy trial, investigating the intervention effect under highly controlled conditions, would probably have had higher attendance rate [67]. Caregivers expressed that the low attendance was mainly due to long traveling distances from their residence to the hospital, work obligations, and social challenges. High prevalence of sharing was reported, though reported as a “small bite”, might compromise the predictive value of the findings. Most caregivers reported using the RUTF as a “supplement” to the habitual diet and not as the “only-diet” consumption, which dilutes the effect of the RUTF products.

Despite the limitations of the trial, the documentation of the effectiveness of a locally developed and produced RUTF compared with a standard RUTF is important. A local product with small fish replacing imported milk-based product has the potential of being part of the solution to improve the treatment of acute malnutrition in Cambodia.

## 5. Conclusions

We showed that neither RUTF was superior in the effectiveness related to weight gain (g/kg/day). A standard RUTF commonly used worldwide was not superior to a locally produced fish-based RUTF with locally available ingredients, and with fish protein as a replacement for milk protein, in the treatment of SAM in Cambodia. To improve the overall effectiveness of the program for the treatment of acute malnutrition, this trial supports that Cambodia needs an integrated MAM–SAM approach in the management of acute malnutrition. In addition, a locally produced RUTF with fish is highly relevant for Cambodia, and it is a potential alternative to the imported, standard milk-based RUTF in the treatment of acute malnutrition.

## Figures and Tables

**Table 1:** Nutritional composition of the RUTFs used in the intervention per 100 g of RUTF.

	NumTrey Paste Only	NumTrey Paste and Wafer	BP-100™	UN Requirements
**Macronutrients**				
Energy (Kcal)	531	506	529	520–550
Protein/Total energy (%)	11.3	9.7	11.1	10–12
Fat/Total energy (%)	56.2	49.6	51.6	45–60
Omega-6/Energy ratio (%)	15	-	-	3–10
Omega-3/Energy ratio (%)	3.6	-	-	0.3–2.5
**Vitamins**				
Vitamin A (mg)	1.1	0.8	0.9	0.8–1.1
Vitamin D (µg)	16.7	11.7	18	15–20
Vitamin E (mg)	19	13.3	27	≥20
Vitamin K (µg)	24.9	17.4	21	15–30
Thiamine (Vitamin B1) (mg)	0.5	0.4	0.5	≥0.5
Riboflavin (Vitamin B2) (mg)	1.6	1.1	1.8	≥1.6
Vitamin C (mg)	56.1	39.3	54	≥50
Vitamin B6 (mg)	0.7	0.5	0.7	≥0.6
Cobalamin (Vitamin B12) (µg)	1.6	1.1	1.6	≥1.6
Folic acid (µg)	355	249	225	≥200
Niacin (mg)	5.8	4.1	5.8	≥5
Pantothenic acid (mg)	3.8	2.7	3	≥3
Biotin (Vitamin B7) (µg)	135	94.5	70	≥60
**Minerals**				
Calcium (mg)	313	219	470	300–600
Sodium (mg)	11.8	8.26	<290	≤290
Potassium (mg)	1104	773	1100	1100–1400
Phosphorus (mg)	424	297	470	300–600
Magnesium (mg)	125	88	110	80–140
Iron (mg)	8.2	5.8	10	10–14
Zinc (mg)	10.8	7.6	12	11–14
Copper (mg)	1.6	1.1	1.5	1.4–1.8
Selenium (µg)	29.3	20.5	25	20–40
Iodine (µg)	112.8	79.0	110	70–140

Abbreviations: UN = United Nations.

**Table 2:** Distributed food ration of BP-100^TM^ and NumTrey based on patient’s weight.

Weight (kg)	Wafers or Bars per Day	
	BP-100^TM^	NumTrey
	Bar	Wafer
3.0–3.4	2	17
3.5–4.9	2.5	20
5.0–6.9	4	27
7.0–9.9	5	40
10.0–14.0	6	53
14.0–19.0	9.5	79
19.0–23.0	13	106
23.0–30.0	16	131
>30.0	200 kcal/kg/day	200 kcal/kg/day

**Table 3:** Baseline characteristics of the patients allocated to BP-100™ or NumTrey based on randomization.

	BP-100™ (61)	NumTrey (60) *	All Patients (121)
Socio-demographic parameters			
Patient age, months	19.7 (12.3)	22.7 (15.1)	21.2 (13.8)
Breastfeeding, % (*n*)	49.2 (30)	45.0 (27)	47.1 (57)
Gender			
Female, % (*n*)	34.4 (21)	48.3 (29)	41.3 (50)
Male, % (*n*)	65.6 (40)	51.7 (31)	58.6 (71)
Child living with family, % (*n*)	96.7 (59)	95.0 (57)	95.9 (116)
Ethnicity			
Khmer, % (*n*)	73.8 (45)	86.7 (52)	80.2 (97)
Main caregiver			
Biological mother, % (*n*)	73.8 (45)	75.0 (45)	74.4 (90)
Nutrition parameters			
Baseline weight, kg	7.32 (1.61)	7.71 (2.02)	7.51 (1.83)
Baseline height, cm	74.8 (9.2)	77.3 (10.8)	76.0 (10.0)
Baseline MUAC, mm	118 (9.0)	119 (7.3)	118 (8.2)
Baseline weight-for-height, z-score	−2.9 (0.7)	−3.0 (0.6)	−3.0 (0.6)
Baseline weight-for-age, z-score	−3.3 (0.9)	−3.2 (0.8)	−3.3 (0.8)
Baseline height-for-age, z-score	−2.3 (1.3)	−2.1 (1.4)	−2.2 (1.4)
Infectious parameters			
HIV positive, % (*n*)	1.6 (1)	-	0.8 (1)
Diarrhea, % (*n*)^1^	31.1 (19)	25.0 (15)	28.1 (34)
Fever, % (*n*)^1^	67.2 (41)	60.0 (36)	63.6 (77)
Lost appetite, % (*n*) ^1^	6.6 (4)	13.3 (8)	9.9 (12)

Data are reported as percentages (*n*) or mean (SD). ^1^ Within one month prior to inclusion in the trial. * For breastfeeding, household information, and infectious parameter, it was not possible to receive information from four patients. Abbreviations: MUAC = mid-upper arm circumference; HIV = human immunodeficiency virus.

**Table 4:** Repeated measurement comparison between BP-100™ and NumTrey on anthropometric parameters using a linear mixed model adjusted for age, gender, and days in the effectiveness and efficacy analysis.

	Effectiveness Analysis					Efficacy Analysis			
	Follow-Up Visits	BP-100™	NumTrey	Difference *^,1^	*p*-Value	BP-100™	NumTrey	Difference *^,1^	*p*-Value
Weight (kg)	Baseline	7.51 ± 0.11 (61)	7.64 ± 0.11 (60)	−0.14 ± 0.16	0.388	7.58 ± 0.91 (60)	7.58 ± 0.90 (61)	0.08 ± 0.09	0.927
	Week 2 follow-up	7.62 ± 0.12 (43)	7.78 ± 0.11 (45)	−0.02 ± 0.06	0.749	7.69 ± 0.94 (41)	7.71 ± 0.91 (47)	0.03 ± 0.06	0.584
	Week 4 follow-up	7.61 ± 0.12 (33)	7.84 ± 0.11 (37)	−0.09 ± 0.07	0.168	7.68 ± 0.95 (34)	7.77 ± 0.94 (36)	0.11 ± 0.07	0.110
	Week 6 follow-up	7.74 ± 0.12 (28)	7.82 ± 0.11 (39)	0.06 ± 0.07	0.399	7.80 ± 0.95 (29)	7.75 ± 0.94 (38)	0.04 ± 0.07	0.524
	Endline	7.79 ± 0.12 (38)	7.94 ± 0.12 (37)	−0.01 ± 0.06	0.843	7.88 ± 0.94 (39)	7.86 ± 0.94 (36)	0.02 ± 0.07	0.779
MUAC (mm)	Baseline	118 ± 1.0 (60)	119 ± 0.1 (60)	−0.24 ± 1.4	0.862	118 ± 0.9 (60)	119 ± 0.9 (60)	−0.11 ± 1.2	0.929
	Week 2 follow-up	121 ± 1.1 (43)	121 ± 1.0 (45)	0.65 ± 1.2	0.574	121 ± 1.1 (41)	121 ± 1.0 (47)	0.86 ± 1.1	0.463
	Week 4 follow-up	122 ± 1.1 (33)	121 ± 1.1 (37)	1.19 ± 1.3	0.342	122 ± 1.1 (34)	121 ± 1.1 (36)	0.76 ± 1.3	0.549
	Week 6 follow-up	122 ± 1.2 (28)	121 ± 1.1 (38)	1.03 ± 1.3	0.429	122 ± 1.1 (28)	121 ± 1.0 (38)	1.25 ± 1.3	0.343
	Endline	123 ± 1.1 (38)	121 ± 1.1 (36)	2.10 ± 1.2	0.090	122 ± 1.0 (38)	122 ± 1.1 (36)	1.68 ±1.3	0.178
WHZ (z-score)	Baseline	−2.98 ± 0.09 (61)	−2.99 ± 0.09 (60)	0.01 ± 0.12	0.905	−2.97 ± 0.08 (60)	−3.00 ± 0.08 (61)	0.04 ± 0.10	0.715
	Week 2 follow-up	−2.81 ± 0.09 (43)	−2.83 ± 0.09 (45)	0.01 ± 0.09	0.952	−2.82 ± 0.09 (41)	−2.82 ± 0.08 (47)	−0.04 ± 0.09	0.689
	Week 4 follow-up	−2.92 ± 0.10 (33)	−2.75 ± 0.09 (37)	−0.19 ± 0.10	0.059	−2.92 ± 0.09 (34)	−2.74 ± 0.09 (36)	−0.22 ± 0.10	0.028
	Week 6 follow-up	−2.70 ± 0.10 (28)	−2.83 ± 0.09 (39)	0.11 ± 0.10	0.255	−2.73 ± 0.09 (29)	−2.81 ± 0.09 (38)	0.04 ± 0.10	0.715
	Endline	−2.68 ± 0.10 (38)	−2.55 ± 0.09 (37)	−0.14 ± 0.10	0.146	−2.64 ± 0.09 (39)	−2.60 ± 0.09 (36)	−0.08 ± 0.10	0.424

Data are reported as mean ± SE (*n*). * Adjusted for baseline values; ^1^ Calculated from baseline to endline with a mean duration of 56 days of treatment. Statistical significance level *p* < 0.05. Abbreviations; MUAC = mid-upper-arm circumference, WHZ = weight-for-height z-score.

## Supplementary Materials

The following are available online at http://www.mdpi.com/2072-6643/10/7/909/s1, Table S1: Repeated measurement comparison between NumTrey and BP-100™ on anthropometric parameters using a linear mixed model adjusted for age, gender, and days in the effectiveness and efficacy analysis.

## Abbreviations

CI	Confidence interval
CMAM	Community-based management of acute malnutrition
HAZ	Height-for-age z-score
HIV	Human immunodeficiency virus
MAM	Moderate acute malnutrition
MUAC	Mid-upper-arm circumference
RUTF	Ready-to-use-therapeutic food
SAM	Severe acute malnutrition
SD	Standard deviation
SE	Standard Error
UNICEF	United Nations Children’s Fund
WAZ	Weight-for-age z-score
WHZ	Weight-for-height z-score
WHO	World Health Organization
