# Supplementary material for: Effectiveness of a Locally Produced, Fish-Based Food Product on Weight Gain among Cambodian Children in the Treatment of Acute Malnutrition: A Randomized Controlled Trial
Source: Nutrients. 2018 Jul 16;10(7):909. doi: 10.3390/nu10070909 (PMC6073612; doi:10.3390/nu10070909)
Supplement: Supplementary file 1 [file nutrients-10-00909-s001.pdf]

Supplementary file

Table 1. Repeated measurement comparison between BP-100™ and NumTrey on anthropometric parameters using a linear mixed model adjusted for age, gender, and days in the effectiveness and efficacy analysis.

		Effectiveness analysis				Efficacy analysis			
	**Follow-up visits**	**BP-100™**	**NumTrey**	**Difference*1**	***p*-Value**	**BP-100™**	**NumTrey**	**Difference*1**	***p*-Value**
Height (cm)	Baseline	76.1 ± 0.5 (61)	76.7 ± 0.5 (60)	−0.7 ± 0.7	0.352	76.5 ± 0.4 (60)	76.3 ± 0.4 (61)	0.1 ± 0.3	0.644
	Week 2 follow-up	76.2 ± 0.5 (43)	76.9 ± 0.5 (45)	− 0.0 ± 0. 2	0.848	76.6 ± 0.4 (41)	76.5 ± 0.4 (47)	0.0 ± 0.2	0.989
	Week 4 follow-up	76.4 ± 0.5 (33)	76.9 ± 0.5 (37)	0.2 ± 0.2	0.332	76.8 ± 0.4 (34)	76.5 ± 0.4 (36)	0.2 ± 0.2	0.309
	Week 6 follow-up	76.4 ± 0.5 (28)	77.1 ± 0.5 (39)	−0.0 ± 0.2	0.906	76.9 ± 0.4 (29)	76.6 ± 0.4 (38)	0.1 ± 0.2	0.532
	Endline	76.7 ± 0.5 (38)	76.9 ± 0.5 (37)	0.5 ± 0.2	0.016	77.1 ± 0.4 (39)	76.5 ± 0.4 (36)	0.4 ± 0.2	0.043
HAZ (z-score)	Baseline	−2.32 ± 0.17 (61)	−2.15 ± 0.17 (60)	−0.17 ± 0.24	0.487	−2.31 ± 0.14 (60)	−2.16 ± 0.14 (61)	−0.15 ± 0.14	0.270
	Week 2 follow-up	−2.31 ± 0.18 (43)	−2.15 ± 0.17 (45)	0.01 ± 0.09	0.876	−2.29 ± 0.15 (41)	−2.17 ± 0.14 (47)	0.03 ± 0.10	0.785
	Week 4 follow-up	−2.26 ± 0.18 (33)	−2.17 ± 0.18 (37)	0.08 ± 0.10	0.417	−2.26 ± 0.15 (34)	−2.17 ± 0.14 (36)	0.05 ± 0.10	0.622
	Week 6 follow-up	−2.30 ± 0.18 (28)	−2.12 ± 0.18 (39)	−0.00 ± 0.11	0.989	−2.27 ± 0.15 (29)	−2.15 ± 0.14 (38)	0.03 ± 0.11	0.767
	Endline	−2.23 ± 0.18 (38)	−2.23 ± 0.18 (37)	0.17 ± 0.10	0.091	−2.25 ± 0.15 (39)	−2.21 ± 0.15 (36)	0.11 ± 0.10	0.282

Data are reported as mean ± SE (*n*). * Adjusted for baseline values.[1] Calculated from baseline to endline with a mean duration of 56 days of treatment. Statistical significance level $p < 0.05$. Abbreviation; HAZ = height-for-age z-score.
